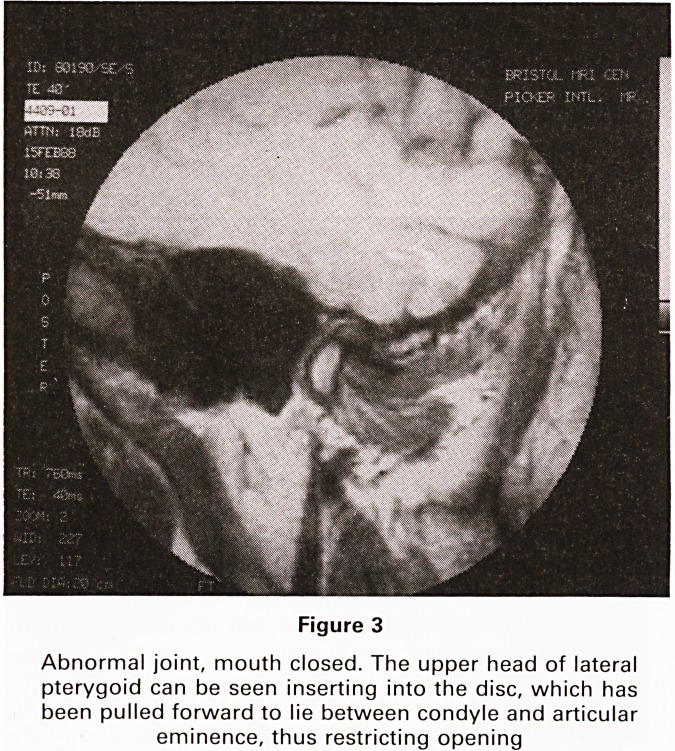# Magnetic Resonance Imaging in the Investigation of Temporo-Mandibular Joint Disorders

**Published:** 1988-05

**Authors:** Martin H Morse


### Magnetic Resonance Imaging in the Investigation of Temporo-Mandibular Joint Disorders

**Martin H Morse**

The large majority of patients with symptoms arising from the TMJ are suffering from TMJ Pain Dysfunction Syndrome, although a small proportion are found to have systemic or degenerative arthritides, or post traumatic conditions. Conservative treatment for Pain Dysfunction Syndrome leads to improvement in many, but there remains a core of patients in whom such measures fail, and who become candidates for surgery. Detailed joint investigation is then mandatory.

The joint anatomy is complicated, with a flexible articular disc attached anteriorly to the lateral pterygoid muscle and anterior condylar neck, posteriorly by fibro-elastic tissue to the skull base and posterior condylar neck, and peripherally to the capsule. The disc separates superior and inferior joint compartments. The articular surfaces, disc, and jont compartments can best be visualised as a series of inverted cups, stacked one upon another. In Pain Dysfunction Syndrome, the dynamics of the disc are usually deranged, with mechanical deformation and displacement producing the limitation of movement and clicks so characteristic of this condition.

Plain film radiography does not allow the disc to be imaged. Computed Tomography is technically difficult if performed in the sagittal plane, and reconstruction from coronal scans results in a considerable radiation dose; in either case the images are of relatively poor quality. The classic investigation is TMJ arthrography, which, when combined with video recording, allows an as yet unrivalled study of disc dynamics; it is, however, invasive, somewhat uncomfortable, and requires a degree of skill both in performance and interpretation.

In recent years, MRI has been used, either in addition to, or as a replacement for, arthrography. Westesson et al. (1987), have compared the MRI appearances with those of Computed Tomography, and with cadaver anatomy, and shown a high degree of correlation. The technique that we employ is to use a T1 weighted spin-echo sequence with a dedicated TMJ coil, and to image the affected joint in closed, and, using a simple inter-dental spacer, open positions. T2 weighted sequences may be used to show joint effusions, but such information rarely adds to the clinical picture, and we do not use them routinely. Comparison with an asymptomatic contralateral joint is, with increasing experience, rarely needed. The total scan time for each position is approximately 6 minutes, and produces images of excellent diagnostic quality. (See Figures 1–3).

In the near future, we hope to utilize fast scanning techniques, which, in combination with a patient operated, graded opening device*, and the necessary software, will produce quasi-cine images, thereby allowing disc dynamics to be studied with the same ease as when using video arthrography.

* Medrad Burnett TMJ Positioning Device.

**ACKNOWLEDGEMENT**

I am grateful to the staff of the Bristol MRI Scanner for their continued help and encouragement.

**Figure 1**

Normal joint, mouth closed. The low signal central portion of the disc lies directly above the condyle (condyle, arrowed)

**Figure 2**

Slightly medial to Figure 1, mouth open. The disc remains superior to the condyle. Both heads of lateral pterygoid are visible inserting into disc and anterior condyle

**Figure 3**

Abnormal joint, mouth closed. The upper head of lateral pterygoid can be seen inserting into the disc, which has been pulled forward to lie between condyle and articular eminence, thus restricting opening

### REFERENCE

WESTESSON, P.-L. KATZBERG, R. W. TALLENTS, R. H. SANCHEZ-WOODWORTH, R. E. SVENSSON, S. A. (1987) CT and MRI of the TMJ, Comparison with autopsy specimens. AJR **148**, 1165–1171.

## Figures and Tables

**Figure 1 f1:**
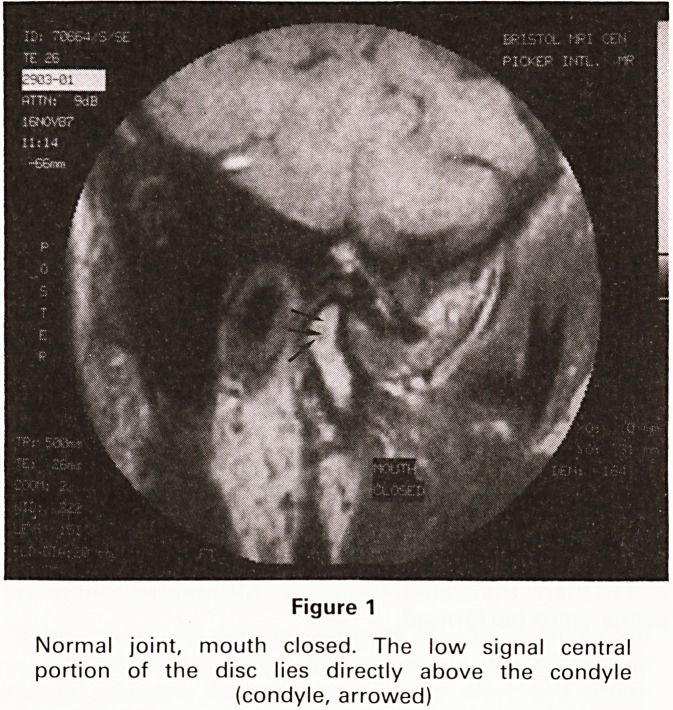


**Figure 2 f2:**
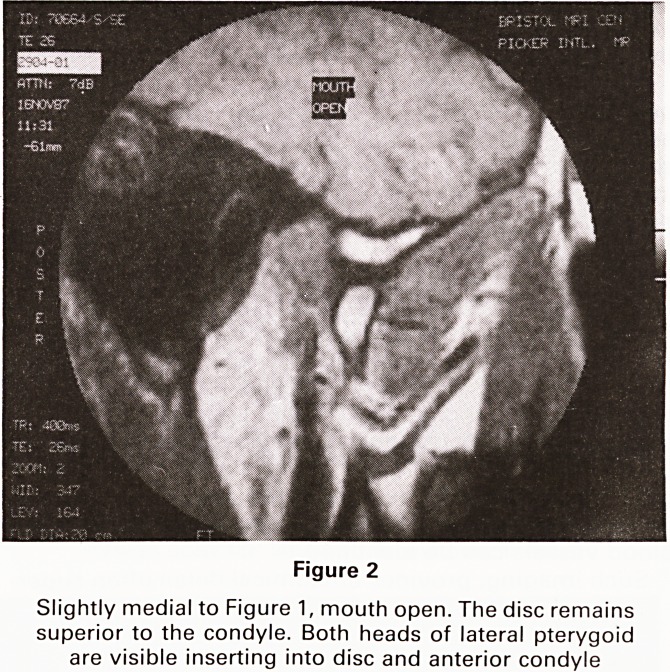


**Figure 3 f3:**